# Systemic Inhibition of CREB is Well-tolerated *in vivo*

**DOI:** 10.1038/srep34513

**Published:** 2016-10-03

**Authors:** Bingbing X. Li, Ryan Gardner, Changhui Xue, David Z. Qian, Fuchun Xie, George Thomas, Steven C. Kazmierczak, Beth A. Habecker, Xiangshu Xiao

**Affiliations:** 1 Program in Chemical Biology, Department of Physiology and Pharmacology, Oregon Health & Science University, 3181 SW Sam Jackson Park Rd, Portland, OR 97239, USA; 2 Knight Cancer Institute, Oregon Health & Science University, 3181 SW Sam Jackson Park Rd, Portland, OR 97239, USA; 3 Department of Pathology, Oregon Health & Science University, 3181 SW Sam Jackson Park Rd, Portland, OR 97239, USA.; 4 Knight Cardiovascular Institute, Department of Medicine, Oregon Health & Science University, 3181 SW Sam Jackson Park Rd, Portland, OR 97239, USA

## Abstract

cAMP-response element binding protein (CREB) is a nuclear transcription factor activated by multiple extracellular signals including growth factors and hormones. These extracellular cues activate CREB through phosphorylation at Ser133 by various protein serine/threonine kinases. Once phosphorylated, it promotes its association with transcription coactivators CREB-binding protein (CBP) and its paralog p300 to activate CREB-dependent gene transcription. Tumor tissues of different origins have been shown to present overexpression and/or overactivation of CREB, indicating CREB as a potential cancer drug target. We previously identified **666-15** as a potent inhibitor of CREB with efficacious anti-cancer activity both *in vitro* and *in vivo*. Herein, we investigated the specificity of **666-15** and evaluated its potential *in vivo* toxicity. We found that **666-15** was fairly selective in inhibiting CREB. **666-15** was also found to be readily bioavailable to achieve pharmacologically relevant concentrations for CREB inhibition. Furthermore, the mice treated with **666-15** showed no evidence of changes in body weight, complete blood count, blood chemistry profile, cardiac contractility and tissue histologies from liver, kidney and heart. For the first time, these results demonstrate that pharmacological inhibition of CREB is well-tolerated *in vivo* and indicate that such inhibitors should be promising cancer therapeutics.

Critical to the success of oncology drug discovery and development programs is to identify an appropriate target whose modulation can lead to selective toxicity in cancer cells without interfering normal cellular homeostasis. In this regard, genetic manipulation strategies including genetic knockdown and ectopic overexpression are powerful approaches to provide critical insights^1^. Cyclic adenosine monophosphate (cAMP) response element-binding protein (CREB) is a transcription factor residing in the cell nucleus to execute the transcriptional responses to extracellular cues including growth factors and hormones^2^. An essential event to activate CREB’s transcriptional response is its phosphorylation at Ser133 by various protein serine/threonine kinases^3^. This phosphorylated CREB (pCREB) can then form a complex with histone acetyl transferases CREB-binding protein (CBP) and its paralog p300 ensuing transcription activation^4^. Among the kinases that can phosphorylate CREB at Ser133 are protein kinase A (PKA), protein kinase B (PKB/Akt), mitogen activated protein kinases (MAPKs) and p90 ribosome S6 kinase (pp90^RSK^)^5^. These protein kinase activities are often over-activated through overexpression or mutation in cancer cells. In normal cells, CREB’s activity is tightly regulated to ensure right response to extracellular cues at right time. Once activated, CREB follows a transcription attenuation phase in the nucleus through dephosphorylation. At least three different phosphatases have been shown to be able to dephosphorylate pCREB. These include protein phosphatase 1 (PP1)^6^, protein phosphatase 2A (PP2A)^7^ and protein phosphatase and tensin homolog (PTEN)^8^. These phosphatases are tumor suppressor proteins that are frequently inactivated or deleted in cancer cells^9^,^10^. The combined activation of CREB kinases and inactivation of CREB phosphatases set CREB in an aberrantly activated state in cancer cells. Indeed, CREB has been shown to be overactivated in multiple solid and liquid cancer tissues^5^,^11^,^12^,^13^,^14^,^15^,^16^. This aberrant overactivation of CREB in cancer cells led to intensive investigation of CREB as a potential target for developing novel cancer therapeutics^5^,^17^,^18^,^19^,^20^,^21^,^22^,^23^,^24^,^25^,^26^.

Numerous studies have shown that knocking down *CREB* in various cancer cells led to inhibition of cancer cell growth both *in vitro* and *in vivo*^5^,^12^,^13^,^15^. Similar genetic manipulations of *CREB* in nontransformed cells were found to be non-toxic^5^,^15^. Notwithstanding good tolerance by normal cells with *CREB* knockdown, complete knockout of *CREB* in mice is perinatal lethal^27^. Furthermore, overexpression of dominant negative CREB (dn-CREB) mutant *CREBS133A* in mouse cardiac myocytes results in dilated cardiomyopathy and heart failure leading to accelerated mortality^28^,^29^,^30^. Given the potential importance of CREB in normal physiology, it is therefore imperative to understand if pharmacological inhibition of CREB is a viable strategy for developing novel cancer therapeutic without deleterious effects in other organs. During the past few years, we have developed a number of cell-permeable first-generation and second-generation small molecule CREB inhibitors^17^,^18^,^19^,^20^,^21^,^22^, some of which have been evaluated for anti-breast cancer efficacy in human xenograft models in mice^18^,^22^. During the investigations of these *in vivo* experiments, we observed no apparent toxicity in the drug-treated mice while displaying significant anti-tumor activity^18^,^22^. Among the second-generation CREB inhibitors, **666-15** (Fig. 1) was the most potent and efficacious one^22^. In this study, we further investigate its specificity against different transcription factors and detail its *in vivo* toxicity in C57BL/6 mice at a therapeutic dose.

## Results

### 666-15 is a specific CREB inhibitor

**666-15** was derived from systematic structure-activity relationship studies of a lead CREB inhibitor naphthol AS-E^17^,^22^. In a cell-based CREB-transcription reporter assay, **666-15** inhibited CREB’s transcription activity with an IC_50_ ~ 80 nM (see also Figure S1A)^22^. We have shown previously that **666-15** did not appreciably inhibit p53-mediated gene transcription and only weakly inhibited NF-κB-mediated gene transcription (IC_50_ = 5290 nM)^22^. To further evaluate the specificity of **666-15**, we investigated its effect on other transcription factors related or unrelated to CREB. CREB activates its transcription through its interaction with CBP/p300 upon phosphorylation at Ser133. To investigate if **666-15** inhibits other transcription factors that interact with CBP/p300 for transcription activation, we tested heterologous transcription activators Gal4-MLL(2840–2858) and Gal4-c-Myb(241–325). MLL(2840–2858) encodes the transcription activation domain from mixed lineage leukemia (MLL) that binds to CBP at an allosteric site to CREB binding site^31^. c-Myb(241–325) is the transcription activation domain from c-Myb that interacts with CBP at the same bindingsite as CREB^31^,^32^. Serum-response factor (SRF) activates gene transcription likely through its interaction with CBP at a different domain than the CREB-binding domain^33^. The Hippo pathway effector TEAD4 (transcriptional enhancer associate domain) activates gene transcription through its interaction with YAP1 (yes-associated protein 1) and its direct interaction with CBP has not been demonstrated^34^. To more specifically investigate **666-15**’s effect on the interaction between the activation domains and transcription coactivators, MLL(2840–2858), c-Myb(241–325) and TEAD4 were all fused in-frame with the DNA-binding domain of yeast Gal4 transcription factor^35^.

**Figure 2:**
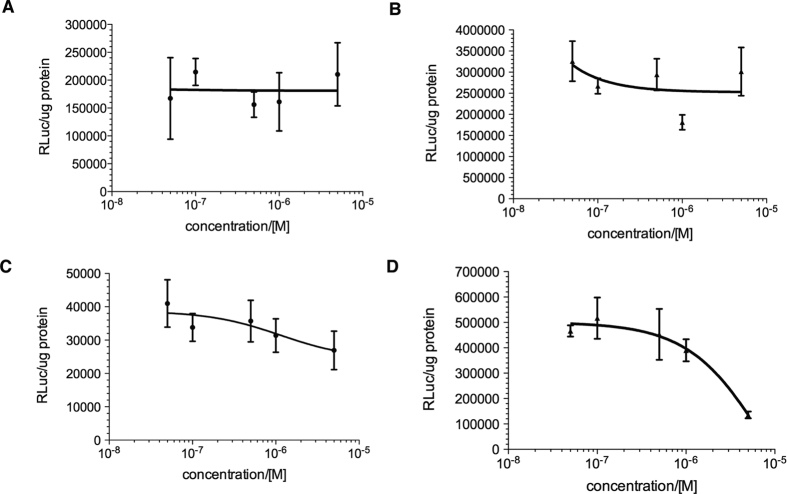
Selectivity of 666-15 against different transcription factors. **666-15** showed little or no inhibition of Gal4-MLL (**A**) Gal4-c-Myb (**B**) Gal4-TEAD4/YAP1 (**C**) and SRF (**D**)-mediated gene transcription. HEK 293T cells were transfected with Gal4-MLL/pG5B (**A**) Gal4-c-Myb/pG5B (**B**) Gal4-TEAD4/YAP1/pG5B (**C**) or SRE-Luc (**D**). Then the transfected cells were treated with increasing concentrations of **666-15** for 5–7 h. The luciferase activity was then measured, normalized to the protein content and expressed as relative luciferase unit (RLuc)/μg protein.

In these transcription reporter assays, HEK293T cells were transfected with Gal4-fusions along with a Gal4-responsive luciferase reporter or SRF-responsive luciferase (SRE-Luc) reporter. For Gal4-TEAD4, coactivator YAP1 was also included for transfection to enhance its transcription activity. Then the cells were treated with increasing concentrations of **666-15**. In contrast to **666-15**’s potent effect on CREB^22^, no inhibition of Gal4-MLL or Gal4-c-Myb-mediated transcription was observed (Fig. 2A,B). Only marginal inhibition (~30%) of Gal4-TEAD4 was seen at high concentrations of **666-15** (5.0 μM) (Fig. 2C). SRF-mediated transcription was inhibited with an IC_50_ ~ 2.0 μM and no inhibition was observed at 100 nM range where significant inhibition of CREB was observed^22^ (Fig. 2D). Together with previous results of **666-15**’s effect on other transcription factors^22^, these data demonstrate that **666-15** had little or no effect on MLL, c-Myb, YAP/TEAD or p53 driven transcription, and only affected NF-κB and SRF driven transcription at concentrations ~100 fold higher than those required for CREB driven transcription.

### Pharmacological concentrations of 666-15 are readily achievable *in vivo*

**Figure 3:**
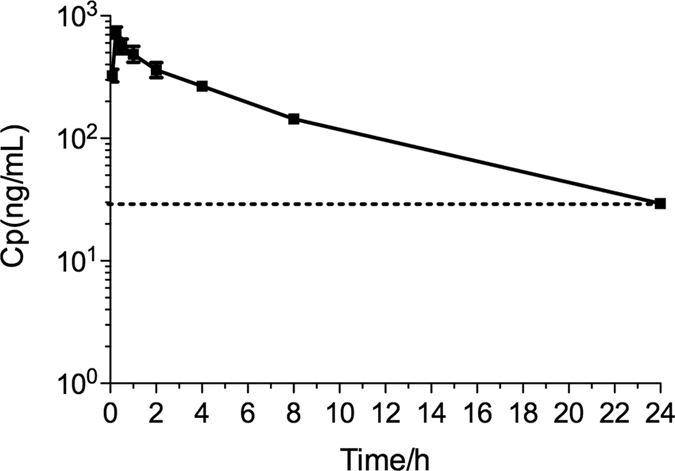
*In vivo* exposure of 666-15. C57BL/6 mice (n = 3) were treated with a single dose of **666-15** at 10 mg/kg by IP injection. Then the blood was collected at different time points and the concentration of **666-15** in the plasma was determined by LC-MS/MS analysis. The dotted line indicates 50 nM of **666-15**.

As a fairly potent and selective CREB inhibitor, **666-15** has been evaluated *in vivo* for its anti-breast cancer effect and efficacious cancer growth inhibitory effect was observed^22^. However, its *in vivo* drug exposure was unclear. In order to evaluate the drug exposure in mice, we treated C57BL/6 mice with a single dose of **666-15** (10 mg/kg) by intraperitoneal (IP) injection, a dose that was used previously for anti-cancer studies^22^. Then the blood was collected at different time points and the plasma drug concentration was measured using tandem mass spectroscopy coupled with liquid chromatography (LC/MS-MS) methods. As shown in Fig. 3, **666-15** was readily bioavailable. The maximal plasma concentration (*C*_*max*_) was 1.26 μM at 15 min post drug administration. What is more important is that pharmacologically relevant concentrations of **666-15** (50-100 nM) for CREB inhibition were detected even 24 h after drug administration. These results demonstrate that *in vivo* exposure of **666-15** is excellent for a once daily (QD) dosing schedule.

### 666-15 does not alter whole body homeostasis *in vivo*

**Figure 4:**
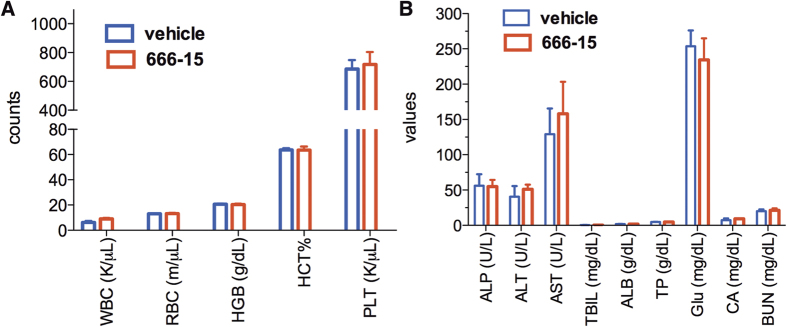
666-15 did not change the CBC or blood chemistry profiles. The mice were treated as described in Materials and Methods. Whole blood (**A**) was used for the CBC and the clinical chemistry profile was performed using plasma (**B**). WBC, white blood cells; RBC, red blood cells; HGB, hemoglobin; HCT, hematocrit; PLT, platelet; ALP, alkaline phosphatase; ALT, alanine aminotransferase; AST, aspartate aminotransferase; TBIL, total bilirubin; ALB, total albumin; TP, total protein; Glu, glucose concentration; CA, calcium; BUN, blood urea nitrogen. All of the parameter comparisons showed *P* > 0.05 by student *t*-test.

**Figure 5:**
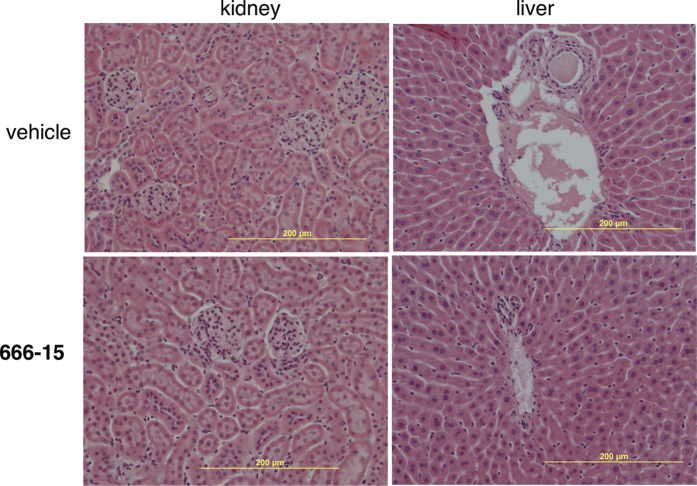
666-15 did not change liver or kidney histologies. The kidneys and livers were collected from the treated mice as described in Materials and Methods section. The kidney and liver slides were stained by hematoxylin and eosin. Representative images are shown.

Previously, we showed that tumor-bearing BALB/c nude mice treated with **666-15** did not experience overt toxicity^22^. In order to investigate its toxicity in more detail, we treated C57BL/6 mice with **666-15** at 10 mg/kg once a day for 5 days a week for 3 weeks, a dosing schedule that was employed before for antitumor studies in BALB/c nude mice^22^. At the end of the treatment, the whole blood was analyzed for complete blood counting (CBC). We observed no difference in all the blood counting parameters between vehicle and **666-15**-treated groups (Fig. 4A). These include white blood cells (WBC), red blood cells (RBC), hemoglobin (HGB), hematocrit (HCT) and platelets (PLT), suggesting that normal hematopoiesis was not altered by **666-15** treatment. The clinical chemistry profiles of the plasma were also analyzed (Fig. 4B). Again we did not find any significant differences in all the parameters analyzed, which include alkaline phosphatase activity (ALP), alanine aminotransferase (ALT), aspartate aminotransferase (AST), total bilirubin (TBIL), total albumin (ALB), total protein (TP), glucose concentration (Glu), calcium (CA) and blood urea nitrogen (BUN). These results suggest that the overall functions of liver, kidney and pancreas are not affected by systemic CREB inhibition with **666-15**. To examine if tissue integrity was affected upon treatment with **666-15**, the livers and kidneys were analyzed by haematoxylin and eosin (H & E) staining. Consistent with the blood chemistry profile, no evidence of damage could be identified from the livers or kidneys in **666-15**-treated mice compared to vehicle-treated ones (Fig. 5).

### 666-15 does not affect cardiac function *in vivo*

**Figure 6:**
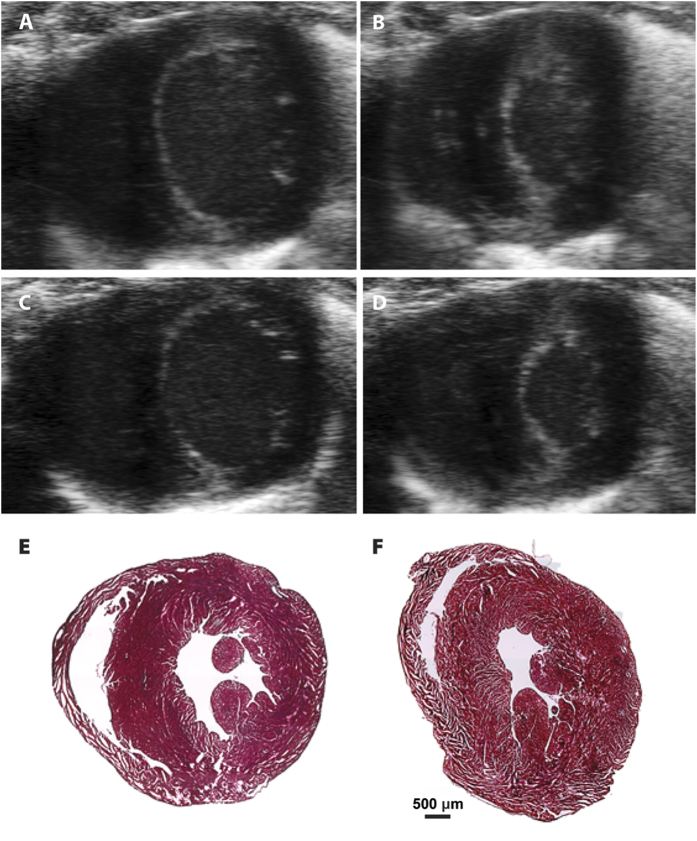
666-15 did not alter cardiac functions in mice. (**A**–**D)** Representative echocardiographic images of the mid-papillary short-axis view in mice treated with vehicle (**A**,**B**) or **666-15** (**C**,**D**) at end-diastole (**A**,**C**) and end-systole (**B**,**D**). (**E**,**F)** Masson’s trichrome stained 10 μm sections of vehicle (**E**) and **666-15** (**F**) treated hearts.

Given the aforementioned phenotypes associated with cardiac function in dn-CREB mice, we investigated in detail the cardiac function of **666-15**-treated mice. Cardiac function was assessed in vehicle and **666-15**-treated mice by non-invasive echocardiography (Fig. 6A–D and Table 1). In contrast to previous studies with expression of dn-CREB in mouse hearts^28^,^29^,^30^, we did not find significant differences in cardiac function between the treatment groups. The parameters measured include heart weight, cardiac output (CO), stroke volume (SV), heart rate (HR), fractional shortening (FS) and ejection fraction (EF) (Table 1). Consistent with these echocardiography measurements, we did not observe any histological or gross pathological difference upon treatment with **666-15** (Fig. 6E,F). Taken together, these results support that systemic inhibition of CREB by pharmacological means does not impair essential cardiac functions.

### 666-15 does not affect basal *tyrosine hydroxylase* (TH) expression

**Figure 7:**
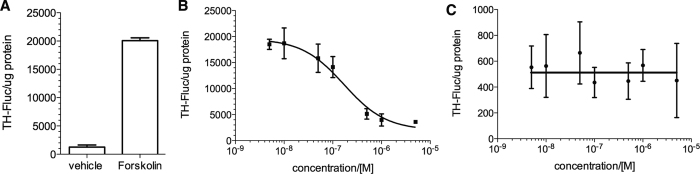
666-15 inhibited forskolin-stimulated, but not basal, TH expression. HEK 293T cells were transfected with TH-Luc. Then the transfected cells were treated as follows: (**A**) The cells were treated with or without forskolin (10 μM). (**B**) The cells were treated with increasing concentrations of **666-15** for 30 min followed by addition of forskolin (10 μM) for 6 h. (**C**) The cells were treated with increasing concentrations of **666-15** for 6.5 h. The luciferase activity was then measured, normalized to the protein content and expressed as TH-Fluc/μg protein.

We further investigated expression of *TH* in superior cervical ganglia (SCG) from the treated mice. TH is the rate-limiting enzyme in the biosynthesis of noradrenaline in neurons and is a CREB target gene^36^,^37^,^38^. No significant change in the expression level of *TH* mRNA was observed (Figure S2). We reasoned that this lack of change in *TH* expression level was due to the notion that basal *TH* expression is less susceptible to inhibition by **666-15** than stimulated expression. To test this hypothesis, we exploited a previously described luciferase reporter construct (TH-Luc)^37^, where the endogenous *TH* promoter (~4.5 kb) was placed upstream of luciferase. Consistent with previous reports^37^, TH expression was significantly enhanced by the treatment of forskolin (Fig. 7A), an activator of CREB-mediated gene transcription^39^. While **666-15** potently inhibited forskolin-stimulated expression of TH (IC_50_ = 0.16 ± 0.11 μM) (Fig. 7B), it did not inhibit basal TH expression (Fig. 7C). Similar results were also obtained when the artificial CREB reporter construct was used (Figure S1B). Therefore, we concluded that basal CREB activity is less susceptible to inhibition by **666-15**, which may contribute to its high tolerance *in vivo*.

## Discussion and Conclusion

As mentioned in the Introduction section, CREB has been shown to be overactivated in cancer tissues from numerous organs, suggesting that CREB is intimately involved in tumorigenesis and tumor maintenance. These results support that targeting CREB represents a novel strategy to develop cancer therapeutics. However, complete knockout of *CREB* in mice was perinatal lethal^27^, which created potential concerns about targeting CREB for developing cancer therapeutics. While it has been well-established that various cancer cells are sensitive to *CREB* knockdown, what has not been demonstrated is if partial inhibition of CREB can be tolerated to maintain normal cellular homeostasis. In this respect, a small molecule inhibitor will be essential because pharmacological inhibitors allow dose adjustment to carefully dial the residual CREB activities.

We have previously shown that both normal epithelial cells and fibroblasts were relatively inert to small molecule-based CREB inhibitors while cancer cells showed exquisite sensitivity^19^,^22^,^40^. Among these CREB inhibitors, **666-15** was the most potent and efficacious one^22^. In this study, we further evaluated **666-15**’s specificity against different transcription factors and found that **666-15** did not appreciably inhibit other transcription factors at concentrations where profound CREB inhibition was observed (Fig. 2 and reference^22^). Specifically, we found Gal4-c-Myb and Gal4-MLL-mediated transcription activities were not inhibited by **666-15** even though similar mechanism of CBP recruitment was necessary for their transcription activation. This is in distinct contrast to other first-generation CREB inhibitors including naphthol AS-E phosphate that inhibits c-Myb’s activity as well^41^,^42^, demonstrating the unique advantages of **666-15** as a novel chemical tool to further investigate the mechanisms of CREB activation and inactivation. Our pharmacokinetic results showed that **666-15** was readily bioavailable to achieve pharmacologically relevant concentrations for CREB inhibition (>50 nM) by once daily IP injection. Importantly, we did not observe any significant functional deterioration of the heart, liver and kidney. These results are in contrast to those obtained in mice with dn-CREB expression in the heart where cardiac hypertrophy, impaired cardiac contractility and premature deaths were observed^28^,^29^,^30^. The difference between dn-CREB and our pharmacological modulation of CREB suggest that either gene dosage of CREB is critical to cardiac myocytes or inhibition of CREB during embryonic development is uniquely disruptive. Whereas dn-CREB expression resulted in constant and perhaps complete inhibition of CREB’s activity, pharmacological administration of **666-15** produced pulsatile inhibition of CREB through drug distribution, metabolism and elimination. This temporally modulated CREB inhibition appears to be well-tolerated in normal tissues. On the other hand, the robust anti-cancer effect seen with **666-15** *in vivo*^22^ supports the notion of cancer cell addiction to CREB^5^. Alternatively, preferential inhibition of stimulated, but not basal, CREB activity by **666-15** may also contribute to our observed *in vivo* safety of **666-15**. Taken all together, these results further demonstrate that pharmacological inhibition of CREB is a viable and promising strategy to develop novel therapeutics perhaps for multiple types of cancers.

## Materials and Methods

### Materials

**666-15** was described before^22^. Throughout the paper, Gal4 refers to Gal4 (1–147) unless specified otherwise. Gal4-MLL was created by fusing yeast Gal4 in-frame to a synthetic oligonucleotide corresponding to human MLL (2840–2858) (DCGNILPSDIMDFVLKNTP) by standard molecular cloning. Gal4-c-Myb (241–325) was a generous gift of Dr. Anders Näär and described before^32^. Gal4-TEAD4 and YAP1 were obtained from Addgene through Dr. Kunliang Guan^34^. pG5B expresses firefly luciferase under the control of Gal4 and was described before^35^. SRE-luciferase was obtained from Promega (Madison, WI).

### Transcription reporter assays

HEK293T cells (ATCC) were routinely cultured in Dulbecco’s modified Eagle medium (DMEM, Life Technologies) supplemented with 10% fetal bovine serum (Hyclone) and non-essential amino acids (Life Technologies). The cells in a well of a 6-well plate were transfected with 1 μg each of the protein-expressing plasmids and 0.5 μg luciferase reporter plasmid by Lipofectamine^2000^ (Life Technologies) following the manufacturer’s instructions. Three hours after transfection, the cells were replated into 96-well plates and were allowed to attach to the bottom of the plates during an overnight incubation. Then the cells were treated with different concentrations of **666-15** for 5–7 h. The luciferase activity was measured by a tube luminometer (Berthold) using luciferase assay reagent (Promega). The protein concentration of the cell lysates in each well was determined using a Protein Assay Dye Reagent (Biorad). The luciferase activity in each well was normalized to the protein content and expressed as RLuc (relative luciferase unit)/μg protein.

### *In vivo* drug treatment

All the procedures for animal handling, care, and the treatment in this study were performed according to the guidelines approved by the Institutional Animal Care and Use Committee (IACUC) of Oregon Health & Science University following the guidelines of the Association for Assessment and Accreditation of Laboratory Animal Care (AAALAC). **666-15** was dissolved in 1% *N*-methylpyrrolidone (NMP), 5% Tween-80 in H_2_O. Female C57BL/6 mice (78–108 days) were treated with vehicle or **666-15** at 10 mg/kg once a day for 5 days a week (Monday-Friday) for 3 weeks (n = 7).

### Whole blood analysis and blood chemistry analysis

A complete blood count was performed using a Vet ABC Hematology Analyzer (Scil Animal Care Company). For the blood chemistry profile, plasma was obtained by centrifuging heparinized whole blood at 1,000× rpm for 5 min at 4 °C. Plasma was stored at −80 °C prior to analysis. The chemistry panel was performed using the comprehensive metabolic panel rotor with an Abaxis Xpress Chemistry Analyzer (Abaxis, Inc., Union City, CA).

### Echocardiography and tissue histology

Echocardiography for left ventricular (LV) function was performed in vehicle and **666-15** treated mice using high-frequency fundamental imaging (Vevo 2100). Mice were sedated with inhaled isoflurane (1.5%), body temperature was maintained at 37 °C, and electrocardiograph (ECG) was continuously recorded throughout the procedure. Images were obtained in the parasternal long-axis plane and parasternal short-axis planes at the midpapillary level. Measurements of LV end-diastolic and end-systolic area (short axis) and end-diastolic and end-systolic length (long axis) were used to measure LV function. Stroke volume was determined using the product of left ventricular outflow tract area and time-velocity integral on pulsed-wave Doppler. Following imaging, the mice were sacrificed. The hearts, livers and kidneys were harvested. The tissues were fixed using 4% paraformaldehyde at room temperature for 1 h. The tissues were then cryosectioned at a thickness of 10 μm and stained using either Masson’s trichrome or hematoxylin and eosin (H&E) staining.

### Real-Time PCR

Superior cervical ganglia (SCG) were removed from the treated mice as representative sympathetic ganglia, and stored immediately in RNAlater (Thermo Fisher). RNA was isolated from individual ganglia using the Ambion RNAqueous micro kit. Total RNA was quantified by OD_260_, and then 200 ng of total RNA was reverse transcribed (RT) and diluted for use. Real-time PCR was performed with ABI TaqMan Universal PCR master mix in the ABI 7500 using ABI pre-validated TaqMan gene expression assays. The mouse gene encoding *tyrosine hydroxylase* (*TH*) was assayed using *glyceraldehyde 3-phosphate dehydrogenase* (*GAPDH*) as a normalization control. For the PCR amplification, 2–4 μl of RT reaction mixture (representing 5 ng of RNA template) was used in a final total volume of 20 μl, and each sample was assayed in duplicate. Standard curves generated with known amounts of RNA from untreated control ganglia, ranging from 0.8–100 ng.

### *In vivo* pharmacokinetics

Female C57BL/6 mice (n = 3) were treated with a single dose of **666-15** at 10 mg/kg by IP injection. Blood was collected at 0.083, 0.25, 0.5, 1, 2, 4, 8, 24 h post drug administration. Blood was collected from each mouse from the submandibular vein (3 time points/mouse). The blood was collected into the EDTA-K2 tubes, and centrifuged at 4,000× rpm for 10 min at 4 °C. The plasma was removed and stored at −80 °C until further analysis. The quantitative LC-MS/MS analyses were conducted using an Agilent 1200 HPLC system coupled to an API 4000 mass spectrometer equipped with an API electrospray ionization (ESI) source. The mobile phases used are a linear gradient of mobile phase A (10 mM ammonium acetate in H_2_O) and mobile phase B (10 mM ammonium acetate in 90% MeOH). The column used was Xtimate C18 2.1 × 30 mm (3 μm) with a flow rate of 0.6 mL/min. The mass spectrometer was operated in ESI positive ion mode and detection of the drugs was performed by multiple reaction monitoring (MRM). The transition of *m*/*z* 584.3 precursor ion to the *m*/*z* 214.1 product ion was used to monitor **666-15** while the transition of *m*/*z* 598.4 precursor ion to the *m*/*z* 228.3 product ion was used for monitoring an internal standard, which is a structural analogue of **666-15** with an extra carbon in the linker^18^. Prior to analysis of the plasma samples, an 8-point standard calibration curve of **666-15** (1, 5, 10, 50, 100, 500, 1000, and 2000 ng/mL) spiked in blank mouse plasma was constructed. Then the plasma samples were analyzed and the concentrations of **666-15** were back-determined from the standard calibration curve.

### Statistical analysis

All the statistical analyses were performed either in Microsoft Excel 2011 or Prism 5. Student *t*-test was used to compare significance. A *P*-value of <0.05 was denoted as significance.

## Additional Information

**How to cite this article**: Li, B. X. *et al*. Systemic Inhibition of CREB is Well-tolerated *in vivo*. *Sci. Rep*. **6**, 34513; doi: 10.1038/srep34513 (2016).

## Supplementary Material

Supplementary Information

## Figures and Tables

**Figure 1:**
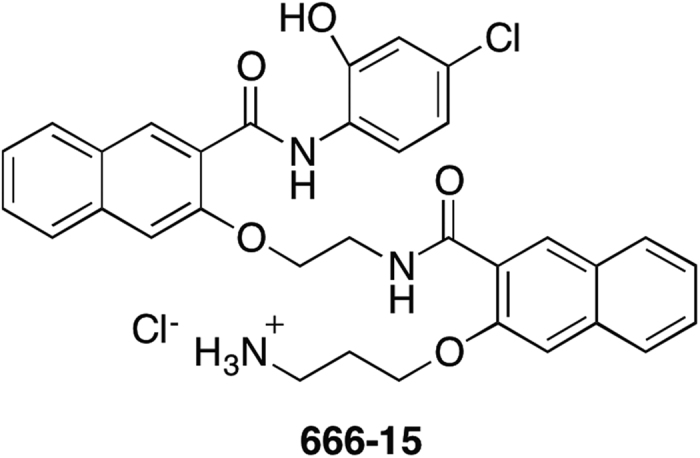
Chemical structure of 666-15.

**Table 1:** Cardiac function measurement of treated mice from echocardiographs^a^.

	BW (g)	HW(mg)	CO (mL/min)	SV (μL)	EF (%)	HR (beats/min)	FS (%)
vehicle	23.77 ± 0.31	143.11 ± 6.19	20.11 ± 0.80	43.29 ± 0.87	61.86 ± 2.29	508.2 ± 10.2	51.05 ± 4.7
**666-15**	23.24 ± 0.28	145.71 ± 5.01	21.89 ± 0.47	42.03 ± 0.56	59.67 ± 1.02	497.6 ± 12.4	50.72 ± 3.1

^a^Values are means ± SEM. BW: body weight; HW: heart weight; CO: cardiac output; SV: stroke volume; HR: heart rate; EF: ejection fraction; FS: fraction shortening. All of the parameter comparisons showed *P* > 0.05 by student *t*-test.
