# Supplementary material for: Systemic Inhibition of CREB is Well-tolerated in vivo
Source: Sci Rep. 2016 Oct 3;6:34513. doi: 10.1038/srep34513 (PMC5046085; doi:10.1038/srep34513)
Supplement: Supplementary Information [file srep34513-s1.pdf]

Supplementary Information

Systemic Inhibition of CREB is Well-tolerated in vivo

Bingbing X. Li,[1,2] Ryan Gardner,[1,2] Changhui Xue,[3] David Z. Qian,[3] Fuchun Xie,[1] George Thomas,[3] Steven C. Kazmierczak,[4] Beth A. Habecker[1,5], and Xiangshu Xiao[1,3,5]*

[1]Program in Chemical Biology, Department of Physiology and Pharmacology, [3]Knight Cancer Institute, [4]Department of Pathology, [5]Knight Cardiovascular Institute, Department of Medicine, Oregon Health & Science University, 3181 SW Sam Jackson Park Rd, Portland, OR 97239, USA

[2]These authors contributed equally.

* To whom correspondence should be addressed: E-mail: xiaoxi@ohsu.edu (XX)

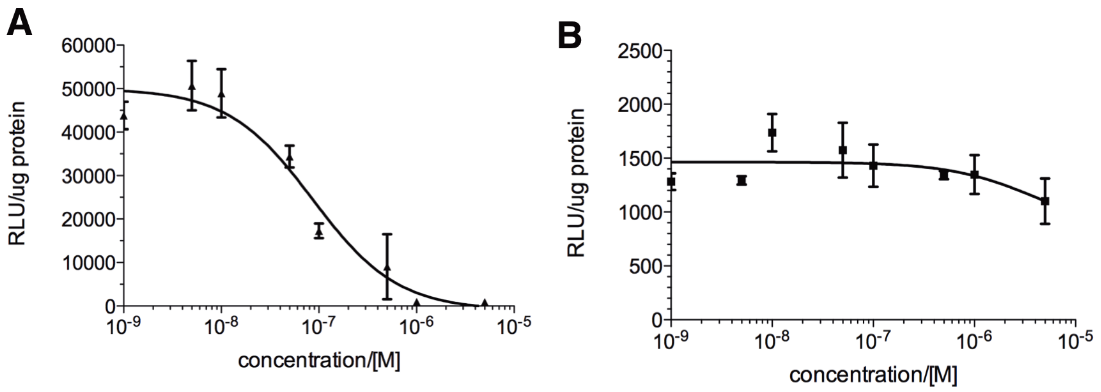

Figure S1. **666-15** potently inhibited CREB-mediated gene transcription in HEK 293T cells. (A) The experiments were performed in the same way as described before.[1] Briefly, HEK 293T cells were transfected with CRE-RLuc construct. Then the cells were treated with increasing concentrations of **666-15** for 30 min followed by treatment with forskolin (10 μM) for 6 h before luciferase measurement. (B) **666-15** did not appreciably inhibit basal CREB activity. The experiments were performed in the same way as in (A) except that forskolin treatment was omitted. The luciferase activity was all normalized to the protein content and expressed as relative luciferase unit (RLU)/μg protein.

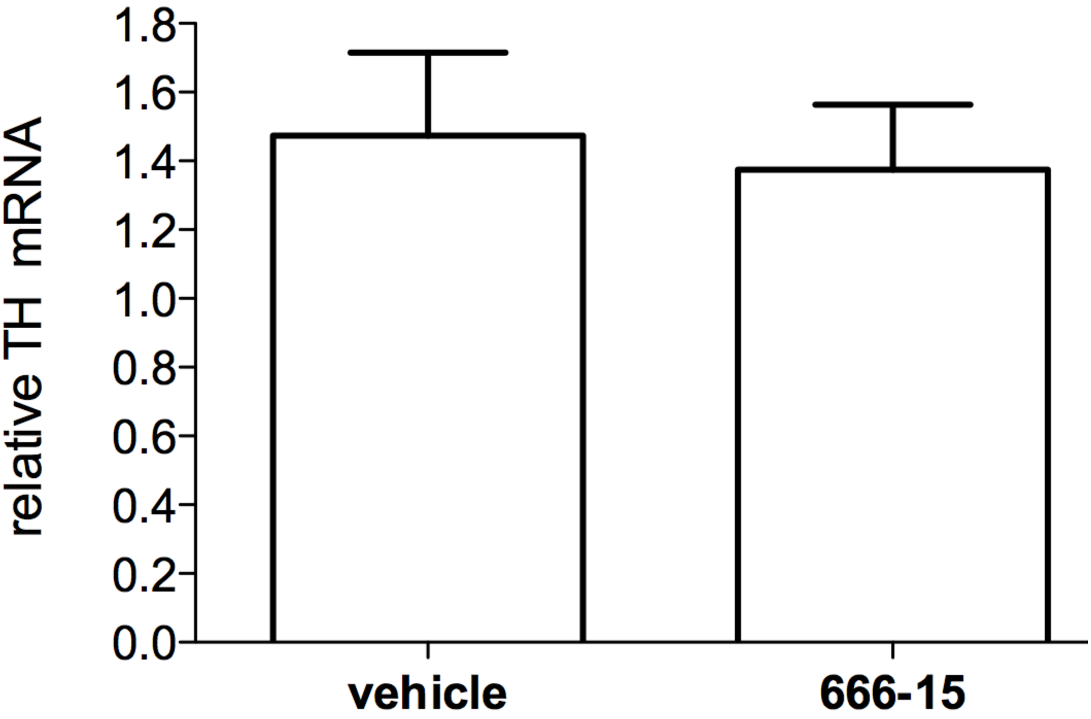

Figure S2. 666-15 did not inhibit basal *TH* expression in superior cervical ganglia *in vivo*. See Methods section for details. $P > 0.05$ by student *t*-test.

References cited:

(1) Xie, F.; Li, B. X.; Kassenbrock, A.; Xue, C.; Wang, X.; Qian, D. Z.; Sears, R. C.; Xiao, X. Identification of a Potent Inhibitor of CREB-Mediated Gene Transcription with Efficacious in Vivo Anticancer Activity. *J. Med. Chem.* **2015**, *58*, 5075-5087.
